# S(+)-ketamine: Current trends in emergency and intensive care medicine

**DOI:** 10.1007/s00508-017-1299-3

**Published:** 2018-01-10

**Authors:** Helmut Trimmel, Raimund Helbok, Thomas Staudinger, Wolfgang Jaksch, Brigitte Messerer, Herbert Schöchl, Rudolf Likar

**Affiliations:** 1 Department of Anaesthesia, Emergency Medicine and Intensive Care and Karl Landsteiner Institute of Emergency Medicine, General Hospital Wiener Neustadt, Corvinusring 3–5, 2700 Wiener Neustadt, Austria; 2 University Hospital for Neurology, Medical University Innsbruck, Innsbruck, Austria; 3 Department of Medicine I, Medical University of Vienna, Vienna, Austria; 4 Department for Anaesthesia, Intensive Care and Pain Medicine, Wilhelminen Hospital of the City of Vienna, Vienna, Austria; 5 Department for Cardiothoracic Anaesthesia, Medical University of Graz, Graz, Austria; 6 AUVA Trauma Hospital, Salzburg, Austria; 7 Department for Anaesthesia and Intensive Care, General Hospital of Klagenfurt, Klagenfurt, Austria

**Keywords:** Ketamine, Analgesia, Neuroprotection, Emergency medicine, Critical care

## Abstract

S(+)-ketamine, the pure dextrorotatory enantiomer of ketamine has been available for clinical use in analgesia and anesthesia for more than 25 years. The main effects are mediated by non-competitive inhibition of the N-methyl-D-aspartate (NMDA) receptor but S(+)-ketamine also interacts with opioid receptors, monoamine receptors, adenosine receptors and other purinergic receptors. Effects on α-amino-3-hydroxy-5-methyl-4-isoxazolepropionic acid (AMPA) receptors, metabotropic glutamate receptors (mGluR) and L‑type calcium chanels have also been described. S(+)-ketamine stimulates the sympathetic nerve system, making it an ideal drug for analgosedation or induction of anesthesia in instable patients. In addition, the neuroprotective properties, bronchodilatory, antihyperalgesic or antiepileptic effects provide interesting therapeutic options. In this article we discuss the numerous effects of S(+)-ketamine under pharmacological and clinical aspects especially for typical indications in emergency medicine as well as intensive care.

From the early 1990s S(+)-ketamine, as the pure dextrorotatory enantiomer of ketamine, has been available for clinical use in analgesia and anesthesia. New and interesting findings concerning the mechanisms of action of this drug, which has a broad spectrum of pharmacologic effects, expanded the field of application over the last decade. Numerous beneficial properties predispose S(+)-ketamine for use as a single agent as well as in combination with other substances for prehospital and hospital-based emergency medicine but more than that it has become an essential part of current treatment concepts for many patients in intensive care, providing specific advantages for those with (traumatic) neurological disorders, bronchospasm, seizures and sepsis. In addition, the considerably reduced side effects of the S(+) enantiomer, especially in terms of psychotropic adverse reactions, have brought new significance compared to racemic ketamine. This article sheds light on the recent value of S(+)-ketamine in these two specific areas, emergency medicine as well as critical care under pharmacological and clinical aspects. In a subsequent review, emphasis is placed on evidence-based as well as expert opinions for the use of this drug in (pediatric) anesthesia, pain medicine and psychiatry (e. g. therapy of treatment-resistant depression).

## Mechanism of action and clinical effects

### Basics

**Fig. 1:**
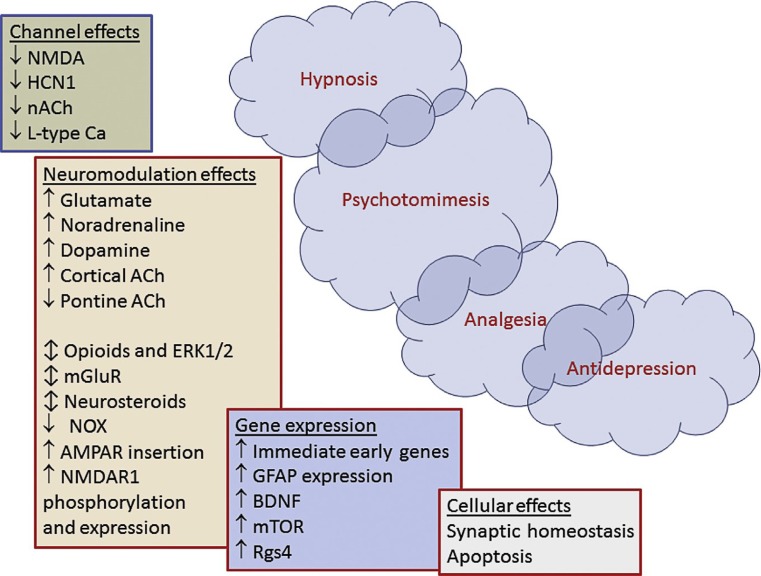
Mechanisms of action of S(+)-ketamine. *NMDA* N-methyl-D aspartate, *HCN1* hyperpolarization-activated cyclic nucleotide channel, *ACh* acetylcholine, *nACh* nicotinergic acetylcholine receptor, *AMPA* α-amino-3-hydroxy-5-methylisoxazole-4-propionic acid, *mGluR* metabotropic glutamate receptor, *ERK1/2* extracellular signal-regulated kinases, *NOX* NADPH oxidase, *BDNF* brain-derived neurotrophic factor, *mTOR* mammalian target of rapamycin, *Rgs4* regulator of G protein signalling 4, *L-type Ca*_*2*_^*+*^ L-type calcium channel, *GFAP* glial fibrillary acidic protein. (Figure used by courtesy of Jamie Sleigh et al. [2] with permission of Elsevier GmbH)

Ketamine is a water and lipid soluble phencyclidine derivative used for anesthesia and sedation. It contains a chiral carbon center, thus enabling two different steric configurations. The two enantiomers have different affinities for the different receptors and consequently somewhat different clinical profiles [1]. S(+)-ketamine influences many cellular processes. Firstly, it is a non-competitive inhibitor of the N‑methyl-D-aspartate (NMDA) receptor via at least two distinct mechanisms, acting both as a channel blocker (effectively shortening the open time) and as an allosteric modulator, reducing the frequency of channel opening. S(+)-ketamine slowly dissociates from the receptor (slow off-rate), even after glutamate has dissociated, and thus causes a persistent blockade. In addition, it is known to interact with opioid receptors, monoamine receptors, adenosine receptors and other purinergic receptors and local anesthetic effects mediated by several ion channels have also been described. The hypnotic effects of S(+)-ketamine are most likely due to the rapid blockade of NMDA and of hyperpolarization-activated cyclic nucleotide-gated cation channels (HCN-1 receptors). Sedative and analgesic effects are probably related to both positive and negative modulation of the cholinergic and aminergic systems, resulting in sensitization of the opioid system combined with enhanced activity of endogenous antinociceptive systems [2]; however, the different systems do not act in isolation but are part of an integrated nervous system with a myriad of interactions on all levels (Fig. 1).

### Immediate and delayed effects

The immediate effects of ketamine include the non-competitive blockade of the NMDA subtype of the glutamate receptor, tonic inhibition of voltage-dependent sodium channels (hypnotic and local anesthetic effects) and blockade of acetylcholine receptors (bronchodilatory effect). Also, at high doses mechanisms involving opiate receptors (δ, μ) lead to potentiation of opiate effects. Effects on α‑amino-3-hydroxy-5-methylisoxazole-4-propionic acid (AMPA) receptors, metabotropic glutamate receptors (mGluR) and L‑type calcium canals have also been described. Another immediate effect is the release of dopamine and noradrenaline. Delayed effects include inhibition of transcription factor expression (c-fos, c‑jun), modulation of the phosphorylation of the NMDA receptor and inhibition of the activation of astrocytes and microglia. The most relevant receptor activities of S(+)-ketamine are depicted in Table 1; [3].

**Table 1:** S_(+)_-ketamine: receptor interactions and clinical effects [3]

Antagonism/inhibition	Agonism/activation
*NMDA receptors*	*Opioid receptors (particularly µ, δ)*
– Dissociative anesthesia	– Central antinociception
– Amnesia	*AMPA receptor*
– Inhibited sensory perception	– Antidepressant
– Analgesia	
*HCN channels*	*GABA*_*A*_ * receptors*
– Hypnosis	– Anesthetic effects
*Calcium channels (L type voltage-dependent)*	
– Negative cardiac inotropy	
– Airway smooth muscle relaxation	
*Voltage-gated sodium channels*	
– Decreased parasympathetic activity	
– Local anesthetic effect	
*BK channels*	
– Analgesic effect on neuropathic pain	

Table used by courtesy of Linda Li and Phillip E. Vlisides [3] with permission of Frontiers Media SA

*NMDA* N-methyl-D-aspartate, *HCN* hyperpolarization-activated cyclic nucleotide, *BK* large conductance potassium channels, *AMPA* α amino-3-hydroxy-5-methyl-4-isoxazolepropionic acid, *GABA*_*A*_*R* $\upgamma$-aminobutyric acid A receptor

### Effects of S(+)-ketamine on other physiological systems

Administration of S(+)-ketamine increases muscle tone and salivation [4]. The swallowing reflex, blinking reflex, coughing reflex and gag reflex remain functional. In the cardiovascular system, ketamine has a dose-dependent stimulatory effect, mainly mediated by the sympathetic nervous system: heart rate, blood pressure and cardiac output all increase. Accordingly, temporary increases in heart rate and blood pressure are cited as common side effects. On the other hand, there is hardly any change in the resistance of the peripheral vasculature. In principle, the central respiratory drive is affected only marginally, although at high doses or with very fast administration of the drug, a depression of breathing may occur. Increased secretion of mucus and a possible increase of resistance in the pulmonary vasculature are also described. Direct effects on smooth muscle cells via voltage-dependent L‑type calcium channels receptors lead to bronchodilation [5]. Besides this, an anti-inflammatory effect has been described [6–9], which may be responsible for the antihyperalgesic effects of ketamine [1]. In sepsis, suppression of the induction of NO synthase and expression of proteins by endotoxins have been observed [10]. The significance of anti-inflammatory effects in daily clinical use of S(+)-ketamine is still under discussion, although experimental data have clearly shown benefits over more than a decade. In addition, current research concerning the antidepressant effects of S(+)-ketamine also revealed that the antinociceptive and anti-inflammatory effects, especially in decreasing inducible Nitric Oxide Synthases (iNOS) are strongly associated with its antidepressant response [11]. No effects of S(+)-ketamine on metabolism, the endocrine system, liver, kidneys, gut function or on blood coagulation are known. In the central nervous system, S(+)-ketamine has cataleptic, potently analgesic and dose-dependent anesthetic effects. Concerns over a drug-induced increase in intracranial pressure using ketamine in the neurosurgical and acute brain injury patients have been present for a long time. Langsjö et al. showed in a prospective observational study during S(+)-ketamine infusion targeted to subanesthetic (150 ng/ml) as well as anesthetic (1500–2000 ng/ml) concentrations by using positron emission tomography (PET) that S(+)-ketamine increases cerebral blood flow (CBF), exceeding the minor changes in metabolic rate of oxygen (CMRO_2_) and glucose metabolic rate (GMR) during anesthesia [12]. Anesthetic doses of ketamine have an effect on cerebral vascular tone; this can increase intracranial pressure in patients with severe traumatic brain injury, especially in combination with hypoventilation and hypertension. This effect can be controlled by normoventilation, which should always be maintained, especially as long as monitoring of intracranial pressure (ICP) is not available. Psychotomimetic effects, which are a major drawback of the racemate and the R(+) enantiomer, are rare at low dosages of S(+)-ketamine (0.125–0.25 mg/kg bodyweight [BW]) but at higher doses they are a frequent problem occurring in up to 12% of cases [13]. At anesthetic doses (0.5–1 mg/kg BW i. v.) S(+)-ketamine gives rise to a characteristic form of dissociative anesthesia: the cataleptic effect leads to an akinetic state with loss of reaction to painful stimuli, but apparently without a complete loss of consciousness. Some patients have their eyes open, some exhibit spontaneous movements, and reflexes such as the corneal reflex, the cough reflex or the swallowing reflex remain functional. Tears and saliva may flow but the patient does not remember the operation or the anesthesia. Occasionally, vivid and possibly unpleasant dreams, sometimes pronounced hallucinations, are triggered; however, these phenomena are considerably less frequent with S(+)-ketamine than with the racemate [14] and can generally be completely suppressed by suitable comedication with propofol or midazolam. Other, more frequent side effects of S(+)-ketamine include nausea or vomiting, impaired vision, dizziness and motor agitation. These effects can, however, almost always be satisfactorily controlled with comedications, such as 5HT3 receptor antagonists or dimenhydrinate. The mechanism of this remains unclear, but could be caused by a dose-dependent interaction of S(+)-ketamine and 5‑HT3 receptor antagonists on this type of receptor [15].

### Neuroprotection

**Fig. 2:**
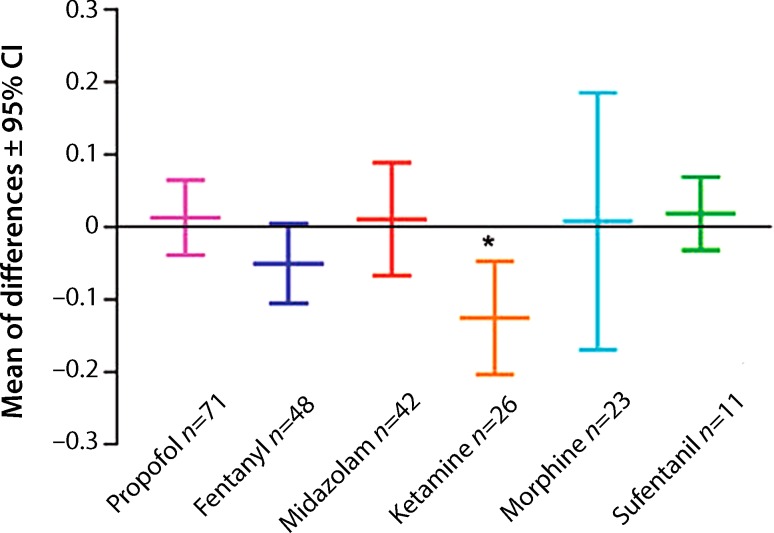
Reduction of relative probability of spreading depolarization (*CI* confidence interval). Figure used by courtesy of D.N. Hertle and J.P. Dreier [20], with permission of Oxford University Press

S(+)-ketamine has a potent and clinically useful neuroprotective effect, because the activation of NMDA receptors is central to the pathophysiological processes that lead from ischemia to apoptosis. Inhibition of the calcium influx into the cell via the antagonistic effect on the NMDA receptor therefore has protective effects on neurons. In cases of severe brain trauma, but also following spontaneous subarachnoid hemorrhage, intracerebral hemorrhage or a space-occupying infarction of the middle cerebral artery, so-called spreading cortical depolarizations can occur (incidence 54–100%). This was first described in 1944 [16] and also plays a key role in the pathophysiology of migraine [17]. These waves of depolarization spread at a rate of 2–7 mm per min across the cortex. They are characterized by a loss of normal ionic homeostasis and especially in cases of severe brain damage, may occur in clusters (>1/h) and lead to neurovascular decoupling, and thus to a secondary phase of brain damage through ischemia and necrosis [18]. Particularly in severe brain trauma, spreading depolarizations, which result in a loss of neuronal activity, are an independent predictor of poor outcome [19]. A retrospective study of 115 patients with acute brain trauma that required surgery investigated the effect of different sedatives and analgesics on spreading depolarizations. S(+)-ketamine yielded a greater reduction of spreading depolarizations than the other substances tested, which were midazolam, fentanyl, propofol and morphine (Fig. 2; [20]). Univariate analysis found a significant correlation between therapy with S(+)-ketamine and the reduction of spreading depolarizations following traumatic brain damage, subarachnoid hemorrhage and malignant hemispheric infarction. In a recently published case report, Schiefecker et al. presented a patient with severe intracerebral bleeding in whom both spreading depolarizations and the concentration of the excitatory neurotransmitter glutamate in the cerebral microdialysate were reduced on treatment with S(+)-ketamine [21]. A possible explanation could be that cortical spreading depolarizations may be suppressed and the cerebral energy utilization is improved by ketamine, which may help maintain the electrochemical ion gradient. It should be mentioned that these findings still have to be proven in prospective randomized controlled trials, and that there is also an ongoing discussion about potential neurotoxic effects of S(+)-ketamine, especially in the context of its anesthetic use in small children [22]. The latter topics will be discussed in more detail in part two of this review.

### Neurotoxicity

Experimental data and animal models raised concern that S(+)-ketamine may be neurotoxic for the developing brain [22, 23]. This seems to be caused by an increase in NMDA receptor NR1 subunit expression, especially when the agent is given repeatedly and in high doses (≥20 mg/kg BW). Consecutive and fulminant influx of Ca^++^ could then lead to apoptotic cell death. Thus, there is on-going discussion about neurofunctional impairment after repeated high-dose ketamine-based anesthesia in (premature) neonates, based on elevated S‑100B levels as well as on clinical findings like a reduction in Bayley scales of infant development (BSID-II) [24]; however, to date no clinical or human data could clearly prove this assumption [22].

### Effect on potential neuronal chronification mechanisms

A further favorable characteristic of S(+)-ketamine is that it helps prevent chronic pain. This effect appears to be due to the suppression of long-term potentiation (LTP). Pain stimuli can cause a sustained overactivation in the sensitive synapses of the pain-conducting C‑fibres that lead into the posterior horn of the spinal cord [25]. The effect is explained in terms of an increased calcium influx into the cells via the NMDA receptors [2], which is largely due to the supraspinal block of the NR2B-NMDA subunit. S(+)-ketamine effectively prevents development of an LTP due to pain stimuli, and this effect has been observed even at doses far below the anesthetic range (0.25 mg/kg BW). This removes a major factor in the emergence of chronic pain and the pain memory.

Opioids promote the excitability of NMDA receptors via activation of µ opioid receptors and in this way they can contribute to a hyperalgesia based on LTP. This can be pre-empted by administering a low dose of S(+)-ketamine before the opioids. At this dose, S(+)-ketamine acts directly on the δ opioid receptor and improves the µ receptor function [2]. It also reinforces the endogenous antinociceptive system independently of the opioid effect by activating the aminergic system (serotonin, noradrenaline) and inhibiting reuptake into the cells. Control of chronic pain is also supported by the effect of S(+)-ketamine on the gene expression cascade linked to pain development. It affects the expression of NMDA receptors, the activation of microglia and astrocytes and synaptic structure and function. Altogether, S(+)-ketamine limits the chronification of pain through all of these mechanisms, but NMDA receptor-related mechanisms play a much stronger role than in acute short-term pain sensations.

## Administration and dosage

S(+)-ketamine can be administered by the following routes: intravenous, intramuscular oral, nasal, intraosseous and even subcutaneous, although only the first two are mentioned in the official Austrian Summary of Product Characteristics (SPC). In children, intramuscular administration should only be used in exceptional cases as this route is painful, stressful and traumatic. Maintenance doses after intramuscular administration should certainly only be given intravenously. Nasal administration can be used for example in burn patients with difficult venous access, preferably using a mucosal atomization device (MAD). The nasal mucosa with its good blood supply guarantees rapid uptake. The better the atomization, the stronger the effect will be. Infusion into the venous system of the superior vena cava avoids a first pass effect and ensures a rapid and titratable action, although there may be variations from individual to individual due to the different characteristics of the vasculature. Slightly higher doses could be used for intranasal (and intraosseous) as for intravenous administration. Bioavailability and the approximate initial dose vary dependent on the route of administration (Table 2; [3, 26]). It is generally recommended to titrate the dose up to the desired clinical effect in order to account for differences in sensitivity between individuals. Analgesia, analgosedation and anesthesia by S(+)-ketamine are dose-dependent [4]. Anesthesia by S(+)-ketamine is rapidly established and relatively short in duration [5]. For analgosedation and anesthesia, combinations of S(+)-ketamine with midazolam or propofol work well and the recommended dosages are shown in Table 3. Due to the fact that a variety of recommendations concerning the dosage of S(+)-ketamine already exist, and older recommendations meant for the racemate are often mismatched, we tried to provide safe and feasible practical recommendations, reflecting recent guidelines and the clinical experience of the authors.

**Table 2:** Bioavailability of S(+)-ketamine via different routes of administration

Route	Bioavailability (in %)	Initial dose (mg/kg BW)		Note
		Analgesia	Anesthesia	
Intravenous^a^	100	0.25	1	Rapid binding to receptor <1 min
Intraosseous	100	0.25	1	Onset of effect similar to i. v.
Intramuscular^a^	93	1	4	Onset of effect after 5–10 min
Oral	16–24	2	–	2.4-fold dose for AUC^b^ similar to i. v.
Nasal	45–50	0.5	–	Not possible to reach anesthetic dose as single agent
Rectal	25–30	1	4	Onset of effect after 10–20 min
Sublingual	24–29	1	–	Not possible to reach anesthetic dose

Recommendations according to Marland et al. [16] and Li and Vlisides [3]. Note: S(+)-Ketamine should *always* be titrated to the required clinical effect.

^a^ Administration recommended in Austrian official Summary of Product Characteristics (SPC)

^b^ *AUC* area under the curve

**Table 3:** Recommended combinations with S(+)-ketamine for continuous analgosedation

0.3–0.5 mg/kg BW/h S_(_+)-ketamine + midazolam 0.03–0.1 mg/kg BW/h
Initial dose approximately 25 mg/h S(+)-ketamine and 2.5 mg/h midazolam in 75 kg adult
Alternative combination with propofol 1–3 mg/kg BW/h (75–150 mg/h in 75 kg adult) higher doses (up to 4 mg/kg BW/h) may be needed in children

## Clinical indications for S(+)-ketamine

Several well-established indications for S(+)-ketamine exist in emergency and intensive care medicine. The substance-specific dissociative anesthesia in which ability to communicate with the patient is retained to a certain degree and, if the substance is not administered too quickly, the absence of depression of breathing function are advantageous in this context, as is the central stimulant effect on the sympathetic nervous system. Especially in shock patients, the latter effect can help to stabilize the circulation. The relaxation of the bronchial muscles and the neuroprotective effect are also positive.

- S(+)-ketamine should therefore be used for induction of anesthesia in patients who are (potentially) hemodynamically unstable [27]. The combination of rapid blood-brain transfer kinetics, sympathomimetic hemodynamic effects and the absence of idiosyncratic side effects are the characteristic advantages in this situation [28]. The substance is also effective in pediatric and geriatric patients and in general well-tolerated [29–31].
- S(+)-ketamine is an excellent choice for induction of anesthesia for potentially unstable cardiac patients, especially combined with midazolam. This was shown for example in patients with septic cardiomyopathy. [32].
- S(+)-ketamine is the drug of choice for induction of anesthesia in patients with bronchospasm [33]. It can protect asymptomatic patients with asthma from developing bronchospasm and it can also effectively relieve bronchospasm in patients who already have respiratory problems before anesthesia [34]. Use of S(+)-ketamine as an analgosedative in patients with refractory asthmatic status, which is not responding to the usual therapeutic options, can reduce the need to initiate mechanical ventilation [5].
- S(+_)_-ketamine can be useful a useful adjunct as a supplementary drug in patients with refractory status epilepticus, due to its anticonvulsive action mediated by the NMDA receptor [35].

## Prehospital emergency and disaster medicine

Based on the pharmacological and clinical characteristics described S(+)-ketamine has to be seen as the anesthetic drug of choice in this field. This is especially the case whenever the options and resources for oxygenation, ventilation, and clinical and instrumental monitoring are limited. The ability to apply S(+)-ketamine intravenously, intranasally, intraosseously, intramuscularly or even subcutaneously, the rapid onset of anesthesia and good controllability the large therapeutic index and the ability to use the drug in non-fasting patients, make it predestined for use in emergency medicine. Excellent stability and long shelf-life make it a useful substance in preparation for major incidents and disasters. Table 4 shows possible dosages in prehospital and hospital-based emergency medicine, depending on the form of administration and the desired effect. Titration of the individual dosage is mandatory, especially in the emergency setting. The spectrum of typical applications (see Table 5 for overview) includes the following conditions in particular:

- *Severe brain trauma*
  S(+)-ketamine is a suitable and safe substance in patients with brain injuries if ventilation and oxygenation are sufficient, even in the hands of less experienced emergency physicians. Contrary to earlier assumptions, therapy with S(+)-ketamine with controlled ventilation does not raise intracranial pressure [36]. As described, due to the blockade of the NDMA receptor, not only an anti-nociceptive effect but also a neuroprotective effect is achieved.
- *Patients in shock*
  Treatment with sedatives, analgesics and anesthetics is often associated with a significant impairment of hemodynamic status [37]. The sympathomimetic effects of S(+)-ketamine can effectively counter this tendency: this is true for analgesia and equally for emergency anaesthesia (usually in the form of rapid sequence induction, RSI). This is particularly important in polytrauma patients, because they often have a brain or spinal trauma and the cerebral and/or spinal perfusion pressure are decisive factors for the prognosis. The hemodynamic stabilization by S(+)-ketamine is independent of the type of shock and is useful in both absolute or relative volume loss and also in cardiogenic shock [28, 32]. If the latter is due to coronary ischemia, although S(+)-ketamine may help to stabilize mean arterial pressure, this may be at the expense of increased afterload and also increased myocardial oxygen demand due to tachycardia; however, in profound shock the sympathomimetic reaction to S(+)-ketamine may also be absent [38].
- *Status asthmaticus, exacerbated chronic obstructive pulmonary disease (COPD)*
  As already mentioned, S(+)-ketamine is the substance of choice for induction of emergency anesthesia in patients with bronchospasm. Another advantage besides the bronchodilatory effect is the fact that it does not trigger histamine release, in contrast to most opioids.
- *Pediatric emergencies*
  Emergency physicians are often somewhat reluctant to administer analgesics or opioids to pediatric patients [39, 40]. Underdosage or even the complete omission of effective pain therapy are quite commonly observed. S(+)-ketamine is a convenient alternative: the analgesic effect is easily titratable in children, and the sedation that begins at higher doses is often desirable. Severe side effects (hypersalivation, nausea, vomiting, central nervous system phenomena, such as double vision, nystagmus, dizziness, agitation and disorientation) are very rare. The incidence of laryngospasm has been estimated at <0.017%, and psychotomimetic reactions (bad trips or arousal reactions such as unpleasant vivid dreams) occur considerably less frequently than in adults [41, 42]. To limit hypersalivation, it is recommended to supplement higher doses of S(+)-ketamine with anticholinergics, such as atropine, or glycopyrrolate, which does not pass the blood-brain barrier.

**Table 4:** Dosage of S(+)-ketamine for different effects (bolus values in mg/kg BW)

Effect	I. v. or I. o.	I. m.	Nasal *(children)*	Rectal *(children)*
*Analgesia* *(Combination with opioids possible)*	Bolus 0.125–0.25	Bolus 0.5 (to 1.0)	Bolus 0.5 (to 2.0)	Bolus 1.0 (to 2.0)
*Analgosedation*	Bolus 0.5 Continuous maintenance dose 0.3–1.5 mg/kg bw/h	Bolus 1.0 (to 2.0)	Bolus 2.0	Bolus 3.0 (to 5.0)
*Induction of (emergency) anesthesia*	Bolus 1.0–1.5	Bolus 2.5 (to 4.0)	–	–
*Maintenance of (emergency) anesthesia*	*Continuous maintenance dose* 1–3 mg/kg BW/h +midazolam 0.1 mg/kg BW/h *or* +propofol 1.0–3.0 mg/kg BW/h			
	*Bolus maintenance* 0.5 every 15–20 min +midazolam bolus (0.05 mg/kg BW) *or* propofol bolus (1.0 mg/kg BW)

**Table 5:** Typical indications for S(+)-ketamine in emergency medicine

*Analgesia*	In trauma patients fractures, burns, soft tissue trauma, etc.
*Analgosedation*	During extrication from vehicles, invasive measures in uncooperative patients
*Anesthesia*	In hypovolemic status and cardiogenic shock
*Asthma*	Induction of anesthesia in asthmatic status, additive to analgosedation in patients with bronchospasm
*Disasters*	Proven worldwide as analgesic and anesthetic in mass casualties, disaster relief and war surgery

Intranasal administration is a practical option that also acts more rapidly. The onset of analgesia is almost similar to intravenous administration, but slightly higher doses are needed (Table 4). Dosage tables that can be accessed on a smartphone are now available, and should help emergency physicians with the procedure [43]. S(+)-ketamine can also be administered intramuscularly, but it should be noted that this route leads to a longer lasting sedation. This route should also be reserved for exceptional cases because of the pain of the injection. Other drugs used in combination with S(+)-ketamine include short-acting benzodiazepines (in hospitals, propofol is most often used [44]), and for severe pain a combination with opioids, especially piritramide, can be recommended. For intravenous analgesia sub-dissociative doses are required and bad trips do not usually occur; however, additional pain relief is desirable and for this reason some emergency physicians prefer not to use additive sedation unless it is needed for anxiolysis. However, as so often in emergency medicine there are no controlled studies only the opinions of experts. In this context, the use of S(+)-ketamine for delayed sequence intubation has to be mentioned [45]. Maintenance of spontaneous breathing during induction of prehospital anesthesia with S(+)-ketamine (1.0–1.5 mg/kg BW) may facilitate a more effective preoxygenation even in agitated patients who do not tolerate conventional methods.

## S(+)-ketamine in intensive care medicine

Analgesia and sedation are core elements of the intensive care therapeutic concept. Pain relief is the top priority. Beyond that, growing attention has been paid to delirium and delirium management, a careful, individualized selection of the substances to use, taking current guidelines [46] into consideration should now be standard. An ideal drug for analgosedation should exhibit rapid onset and cessation of action for optimum control, be freed from active metabolites and cardiovascular side effects and the lowest possible tendency to cause delirium. The pharmacokinetic and pharmacodynamic properties of S(+)-ketamine already described come very close to these ideals, so that it plays a central role in a state of the art, analgesia-based sedation concept both for short-term and longer-term use and in patients with a number of particular conditions [47].

### Analgosedation in neurotrauma

Analgosedation in patients with brain trauma is aimed primarily at preventing elevation of intracranial pressure due to pain or agitation and at the facilitation of mechanical ventilation [48]. As previously described, S(+)-ketamine also has potential neuroprotective effects based on the reduction of glutamate excitotoxicity, anti-inflammatory action, e.g. reduced microglia activation, reduction of inflammatory cytokines, such as tumor necrosis factor (TNF) and interleukin 6 (IL-6) and the reduction of spreading depolarizations [49]. Patients with brain injuries also benefit from the antioxidative [50] and anticonvulsive [50–53] effects of S(+)-ketamine. Due to its stabilizing effects on the circulation, it also helps to limit hypotension and therefore, potentially, hypotension-related secondary brain damage [50]. No adverse effects on intracranial pressure have been detected when using S(+)-ketamine in combination with GABA receptor agonists, such as benzodiazepines and barbiturates to suppress the excitatory component, even in patients with intracranial hypertension [46]. In brain trauma, the combination of S(+)-ketamine and midazolam yields better cardiovascular stability. The use of vasopressors is lower compared to sufentanil/midazolam and a better fluid balance is achieved [48]. In neurological intensive care patients, S(+)-ketamine can be used to induce anesthesia as a bolus of 1.0 mg/kg BW combined with midazolam at 0.1 mg/kg BW. The maintenance dose is 0.5–3 mg/kg BW/h of S(+)-ketamine plus 0.1–0.5 mg/kg BW/h of midazolam.

### S(+)-ketamine in chronic obstructive pulmonary disease (COPD) and asthma

Under these conditions, S(+)-ketamine is suitable both for induction of anesthesia for intubation and for continuous analgosedation of already intubated patients. Normally, the latter is done in combination with propofol and high-dose magnesium. Also in patients with obstructive conditions undergoing non-invasive ventilation, S(+)-ketamine can be useful in combination with low-dose propofol or midazolam for short-term sedation to alleviate agitation and mask intolerance, especially at the beginning of therapy, because it does not affect the respiratory drive.

### S(+)-ketamine in refractory status epilepticus

Taking advantage of its anticonvulsive effect, S(+)-ketamine is increasingly being used to treat refractory status epilepticus (RSE) [35, 54, 55]. This condition is characterized by failure to respond to first-line (usually benzodiazepine) and second-line anti-seizure medication (e.g. antiepileptic drug, propofol or barbiturates), ideally under monitoring by electroencephalogram (EEG). Ongoing seizure activity causes internalization, and therefore loss, of GABA-A receptors from the synaptic membrane. Therefore, some medications (e. g. benzodiazepines) lose their efficacy. The effect of S(+)-ketamine can be explained by the fact that seizure activity causes an increase in the number of excitatory receptors (NMDA) in the synaptic membrane. Thus, S(+)-ketamine may be an alternative to general anesthesia especially in prolonged, persistent, or recurrent seizures. Some experts recommend the use of ketamine even as early as ≥30 min after onset [56]. The administration and dosage are similar to those used for intensive care patients (1.5 mg/kg BW i. v. every 3–5 min until seizures stop, max. 4.5 mg/kg BW). Initial infusion is 1.2 mg/kg BW/h, maintenance dose is 0.3–7.5 mg/kg BW/h [56], but has been described to be as high as 10 mg/kg BW/h in a retrospective review of medical records [57].

### S(+)-ketamine and sepsis

In septic shock, S(+)-ketamine, in combination with propofol and if needed, opioids, has several advantages because of its betamimetic and alphamimetic properties:

- Catecholamine release from the adrenal gland [58].
- Cortisone release (in septic shock, two thirds of patients have a relative adrenal insufficiency with inadequate levels of cortisone) [59–61].
- These effects result in a saving in the amount of vasopressor(s) needed [59].
- Possibly, immunomodulation also plays a role but only experimental data are available on this [62, 63].
- Avoidance of additional, opioid-induced inhibition of gut mobility in sepsis-induced intestinal paralysis [64].
- Relatively early return of a relevant level of spontaneous breathing due to the almost complete absence of depression of respiratory drive.

### S(+)-ketamine for long-term sedation in intensive care

S(+)-ketamine has a special value as an additive drug in analgosedation of intensive care patients and if possible, should be part of the treatment concept from the onset. In patients sedated for longer periods it is regularly used in combination with other substances [47], particularly with sedatives such as midazolam, propofol and clonidine. It is also often combined with opioid analgesics such as remifentanil. Burns patients in particular benefit from this therapy, especially with respect to therapeutic manipulations, such as changing of dressings [30]. The opioid-saving effect is especially beneficial in patients who need to be deeply sedated for longer periods, because they frequently develop tolerance when receiving opioids and midazolam. Addition of S(+)-ketamine can enable a dose reduction of both these substances and also make the withdrawal process easier. In fact, there is a lot to be said for combining S(+)-ketamine with continuous opioid treatment from the outset, in order to prevent the development of hyperalgesia and tolerance [65, 66]. Dosage for long-term analgosedation with S(+)-ketamine essentially follows the same scheme as shown in Table 4 and should be within the range of 0.25 mg/kg BW/h to a maximum of 1.5 mg/kg BW/h.

## Substance abuse

Because of its psychedelic action, ketamine also has the potential to be misused as a recreational drug [66, 67]. This has been known for a long time; however, the addiction potential of the S‑enantiomer is significantly lower than that of the racemate because of its weaker psychotomimetic effects. Despite this, many internet pages can be found offering sometimes astonishingly detailed ‘recommendations’ on how to use it. Preclinical therapy of ketamine abuse concentrates mainly on symptomatic control of the overstimulation of the sympathetic nervous system [68]. Hallucinations comprising extracorporeal perception or the ability to fly can lead to life-threatening events, as well as combined drug abuse.

## Summary

- S(+)-ketamine exerts its effects mainly via the non-competitive blockade of the NMDA receptor. Additionally, interactions with opioid receptors, monoamine receptors, adenosine receptors, other purinergic receptors and cholinergic receptors have been described. S(+)-ketamine is used for analgesia, analgosedation and anesthesia. It is a potent analgesic, precludes opioid-induced hyperalgesia. The sedative and anesthetic action of the drug is dose-dependent.
- Specific effects of S(+)-ketamine include stimulation of the circulation, bronchodilation, inhibition of inflammation and the characteristic dissociative anesthesia. Muscle tone and salivation increase; swallowing reflexes remain. Pronounced neuroprotective effects, arising from the inhibition of calcium influx into the cells, have been demonstrated. Studies show a significant reduction of spreading depolarizations (which are associated with further neuronal degeneration) following brain trauma.
- S(+)-ketamine is useful for preventing chronification of pain based on long-term potentiation (long-lasting overactivation at sensitive synapses of pain-conducting C fibers).
- In prehospital emergency medicine, S(+)-ketamine is an excellent option for analgosedation or induction of anesthesia for all patients with impaired hemodynamics. This is especially true for patients with brain trauma who have adequate ventilation and oxygenation: S(+)-ketamine can be used to ensure sufficient cerebral perfusion pressure. Reports of using S(+)-ketamine in children are also generally positive.
- Well-established indications for using S(+)-ketamine include intravenous induction of anesthesia, also in other emergency situations, such as status asthmaticus, (prehospital) anesthesia of patients in cardiogenic shock, analgosedation of patients with burns and analgosedation in the context of mass casualty incidents.
- In intensive care medicine, S(+)-ketamine is a well-established substance for analgosedation in general; in particular, patients with (traumatic) brain damage can benefit from its additional neuroprotective and anticonvulsive effects and from the reduction in free radicals. At constant pCO_2_ and mean arterial pressure, the therapy does not cause any rise in intracranial pressure. S(+)-ketamine is also a useful adjunct in refractory status epilepticus.
- In treatment of patients requiring long-term sedation, S(+)-ketamine in combination with other substances contributes to a reduction in opioid use and a reduction in the effort needed to manage withdrawal. The antihyperalgesic action is particularly relevant in view of tolerance and related side effects of opioids.

## References

[1] Persson J (2010). Wherefore Ketamine?. Curr Opin Anaesthesiol.

[2] Sleigh J, Harvey M, Voss L, Denny B (2014). Ketamine—More mechanisms of action than just NMDA blockade. Trends Anaesth Crit Care.

[3] Li L, Vlisides PE (2016). Ketamine: 50 years of modulating the mind. Front Hum Neurosci.

[4] Kolawole IK (2001). Ketamine hydrochloride: a useful but frequently misused drug. Niger J Surg Res.

[5] Goyal S, Agrawal A (2013). Ketamine in status asthmaticus: a review. Indian J. Crit. Care. Med..

[6] Taniguchi T, Shibata K, Yamamoto K (2001). Ketamine inhibits endotoxin-induced shock in rats. Anesthesiology.

[7] Yu M, Shao D, Liu J, Zhu J, Zhang Z, Xu J (2007). Effects of ketamine on levels of cytokines, NF-κB and TLRs in rat intestine during CLP-induced sepsis. Int Immunopharmacol.

[8] Yu M, Shao D, Yang R, Feng X, Zhu S, Xu J (2007). Effects of ketamine on pulmonary inflammatory responses and survival in rats exposed to polymicrobial sepsis. J Pharm Pharm Sci.

[9] Gokcinar D, Ergin V, Cumaoglu A, Menevse A, Aricioglu A (2013). Effects of ketamine, propofol, and ketofol on proinflammatory cytokines and markers of oxidative stress in a rat model of endotoxemia-induced acute lung injury. Acta Biochim Pol.

[10] Yoon SH (2012). Concerns of the anesthesiologist: anesthetic induction in severe sepsis or septic shock patients. Korean J Anesthesiol.

[11] do Vale EM, Xavier CC, Nogueira BG, Campos BC, Aquino PEA, Costa RO, Barros Viana GS (2016). Antinociceptive and antiinflammatory effects of Ketamine and the relationship to its antidepressant action and GSK3 inhibition. Basic. Clin. Pharmacol. Toxicol..

[12] Långsjö JW, Maksimow A, Salmi E, Kaisti K, Aalto S, Oikonen V, Parkkola R (2005). S-ketamine anesthesia increases cerebral blood flow in excess of the metabolic needs in humans. Anesthesiology.

[13] Annetta MG, Iemma D, Garisto C, Tafani C, Proietti R (2005). Ketamine: new indications for an old drug. Curr Drug Targets.

[14] Kress HG (1997). Wirkmechanismen von Ketamin. Anaesthesist.

[15] Suzuki T, Aoki T, Kato H, Yamazaki M, Misawa M (1999). Effects of the 5‑HT 3 receptor antagonist ondansetron on the ketamine-and dizocilpine-induced place preferences in mice. Eur. J. Pharmacol..

[16] Leão AAP (1944). Spreading depression of activity in the cerebral cortex. J Neurophysiol.

[17] Dreier JP, Reiffurth C (2015). The stroke-migraine depolarization continuum. Neuron.

[18] Dreier JP, Fabricius M, Ayata C, Sakowitz O, Shuttleworth CW, Dohmen C (2016). Guidelines of the COSBID study group on recording and analysis of spreading depolarizations in the clinic. J. Cereb. Blood. Flow Metab..

[19] Hartings JA, Watanabe T, Bullock MR, Okonkwo DO, Fabricius M, Woitzik J (2011). Spreading depolarizations have prolonged direct current shifts and are associated with poor outcome in brain trauma. Brain.

[20] Hertle DN, Dreier JP, Woitzik J, Hartings JA, Bullock R, Okonkwo DO, Shutter LA, Vidgeon S, Strong AJ, Kowoll C, Dohmen C, Diedler J, Veltkamp R, Bruckner T, Unterberg AW (2012). Sakowitz OW and for the cooperative study of brain injury depolarizations (COSBID). Effect of analgesics and sedatives on the occurrence of spreading depolarizations accompanying acute brain injury. Brain.

[21] Schiefecker AJ, Beer R, Pfausler B, Lackner P, Broessner G, Unterberger I (2015). Clusters of cortical spreading depolarizations in a patient with intracerebral hemorrhage: a multimodal neuromonitoring study. Neurocrit Care.

[22] Yan J, Jiang H (2014). Dual effects of ketamine: neurotoxicity versus neuroprotection in anesthesia for the developing brain. J Neurosurg Anesthesiol.

[23] Zou X, Patterson TA, Sadovova N, Twaddle NC, Doerge DR, Zhang X, Wang C (2009). Potential neurotoxicity of ketamine in the developing rat brain. Toxicol Sci.

[24] Yan J, Li YR, Zhang Y, Lu Y, Jiang H (2014). Repeated exposure to anesthetic ketamine can negatively impact neurodevelopment in infants: a prospective preliminary clinical study. J Child Neurol.

[25] Benrath J, Brechtel C, Stark J, Sandkühler J (2005). Low dose of S(+)-ketamine prevents long-term potentiation in pain pathways under strong opioid analgesia in the rat spinal cord in vivo. Br J Anaesth.

[26] Marland S, Ellerton J, Andolfatto G, Strapazzon G, Thomassen O, Brandner B, Paal P (2013). Ketamine: use in anesthesia. Cns Neurosci Ther.

[27] Roessler M (2016). Prähospitale Notfallnarkose beim Erwachsenen. Notf. Rettungsmed..

[28] Morris C, Perris A, Klein J, Mahoney P (2009). Anaesthesia in haemodynamically compromised emergency patients: Does ketamine represent the best choice of induction agent?. Anaesthesia.

[29] Green SM, Roback MG, Kennedy RM, Krauss B (2011). Clinical practice guideline for emergency department Ketamine dissociative sedation: 2011 update. Ann Emerg Med.

[30] Gündüz M, Sakallı Ş, Güneş Y, Kesiktaş E, Özcengiz D, Işık G (2011). Comparison of effects of ketamine, ketamine-dexmedetomidine and ketamine-midazolam on dressing changes of burn patients. J Anaesthesiol Clin Pharmacol.

[31] O’Hara D, Ganeshalingam K, Gerrish H, Richardson P (2014). A 2 year experience of nurse led conscious sedation in paediatric burns. Burns.

[32] De la Grandville B, Arroyo D, Walder B (2012). Etomidate for critically ill patients. Con: Do you really want to weaken the frail?. Eur J Anaesthesiol.

[33] Stoelting RK, Hillier SC, Stoelting RK, Hillier SC (2006). Nonbarbiturate intravenous anaesthetic drugs. Pharmacology and physiology in anaesthetic practice.

[34] Corssen G, Gutierrez J, Reves JG, Huber FC (1972). Ketamine in the anesthetic management of asthmatic patients. Anesth. Analg..

[35] Zeiler FA, Teitelbaum J, Gillman LM, West M (2014). NMDA antagonists for refractory seizures. Neurocrit Care.

[36] Zeiler FA, Teitelbaum J, West M, Gillman LM (2014). The ketamine effect on ICP in traumatic brain injury. Neurocrit Care.

[37] Heffner AC, Swords D, Kline JA, Jones AE (2012). The frequency and significance of postintubation hypotension during emergency airway management. J Crit Care.

[38] Miller M, Kruit N, Heldreich C, Ware S, Habig K, Reid C, Burns B (2016). Hemodynamic response after rapid sequence induction with ketamine in out-of-hospital patients at risk of shock as defined by the shock index. Ann Emerg Med.

[39] Rutkowska A, Skotnicka-Klonowicz G (2015). Prehospital pain management in children with traumatic injuries. Pediatr Emerg Care.

[40] Samuel N, Steiner IP, Shavit I (2015). Prehospital pain management of injured children: a systematic review of current evidence. Am J Emerg Med.

[41] Sherwin TS, Green SM, Khan A, Chapman DS, Dannenberg B (2000). Does adjunctive Midazolam reduce recovery agitation after Ketamine sedation for pediatric procedures? A randomized, double-blind, placebo-controlled trial. Ann Emerg Med.

[42] Wathen JE, Roback MG, Mackenzie T, Bothner JP (2000). Does Midazolam alter the clinical effects of intravenous Ketamine sedation in children? A double-blind, randomized, controlled, emergency department trial. Ann Emerg Med.

[43] Kinder Notfall Tabelle Pro, 2015, planerapps e.U., https://play.google.com/store/apps/details?id=com.planerapps.kinderNotfallTabellePro. Accessed 17 Aug 2017.

[44] Thomas MC, Jennett-Reznek AM, Patanwala AE (2011). Combination of Ketamine and Propofol versus either agent alone for procedural sedation in the emergency department. Am J Health Syst Pharm.

[45] Weingart SD, Trueger NS, Wong N, Scofi J, Singh N, Rudolph SS (2015). Delayed sequence intubation: a prospective observational study. Ann Emerg Med.

[46] DGAI, DIVI (2015). S3-Leitlinie: Analgesie, Sedierung und Delirmanagement in der Intensivmedizin. AWMF-Registernummer: 001/012.

[47] Herzer G, Mirth C, Illievich U, Voelckel W, Trimmel H (2017). Analgosedation of adult patients with elevated intracranial pressure. Survey of current clinical practice in Austria. Wien Klin Wochenschr.

[48] Bourgoin A, Albanèse J, Wereszczynski N, Charbit M, Vialet R, Martin C (2003). Safety of sedation with ketamine in severe head injury patients:comparison with sufentanil. Crit Care Med.

[49] Chang LC, Raty SR, Ortiz J, Bailard NS, Mathew SJ (2013). The emerging use of Ketamine for anesthesia and sedation in traumatic brain injuries. CNS Neurosci. Ther..

[50] Sih K, Campbell SG, Tallon JM, Magee K, Zed PJ (2011). Ketamine in adult emergency medicine: controversies and recent advances. Ann Pharmacother.

[51] Dorandeu F (2017). Ketamine for the treatment of (super) refractory status epilepticus? Not quite yet. Expert Rev Neurother.

[52] Fang Y, Wang X (2015). Ketamine for the treatment of refractory status epilepticus. Seizure.

[53] Ilvento L, Rosati A, Marini C, L’Erario M, Mirabile L, Guerrini R (2015). Ketamine in refractory convulsive status epilepticus in children avoids endotracheal intubation. Epilepsy Behav.

[54] Gaspard N, Foreman B, Judd LM, Brenton JN, Nathan BR, McCoy BM, Al-Otaibi A, Kilbride R, Sánchez Fernández I, Mendoza L, Samuel S, Zakaria A, Kalamangalam GP, Legros B, Szaflarski JP, Loddenkemper T, Hahn CD, Goodkin HP, Claassen J, Hirsch LJ, LaRoche SM, Critical Care EEG Monitoring Research Consortium (2013). Intravenous ketamine for the treatment of refractory status epilepticus: a retrospective multicenter study. Epilepsia.

[55] Borris DJ, Bertram EH, Kapur J (2000). Ketamine controls prolonged status epilepticus. Epilepsy Res.

[56] Grover EH, Nazzal Y (2016). Hirsch LJ Treatment of convulsive status epilepticus. Curr Treat Options Neurol.

[57] Trinka E, Höfler J, Leitinger M, Brigo F (2015). Pharmacotherapy for status epilepticus. Drugs.

[58] Adams HA, Werner C (1997). Vom Razemat zum Eutomer: (S)-Ketamin—Renaissance einer Substanz?. Anaesthesist.

[59] Adams HA (1997). Endokrine Reaktionen nach S‑(+)-Ketamin. Anaesthesist.

[60] Annane D, Sébille V, Charpentier C, Bollaert PE, François B, Korach JM, Capellier G, Cohen Y, Azoulay E, Troché G, Chaumet-Riffaut P, Bellissant E (2002). Effect of treatment with low doses of Hydrocortisone and Fludrocortisone on mortality in patients with septic. JAMA.

[61] Annane D, Maxime V, Ibrahim F, Alvarez JC, Abe E, Boudou P (2006). Diagnosis of adrenal insufficiency in severe sepsis and septic shock. Am. J. Respir. Crit. Care. Med..

[62] Lange M, Bröking K, van Aken H, Hucklenbruch C, Bone HG, Westphal M (2006). Einsatz von Ketamin bei Sepsis und systemischen Entzündungsreaktionen. Anaesthesist.

[63] Beilin B, Rusabrov Y, Shapira Y, Roytblat L, Greemberg L, Yardeni IZ, Bessler H (2007). Low-dose ketamine affects immune responses in humans during the early postoperative period. Br. J. Anaesth..

[64] Freye E, Knüfermann V (1994). Keine Hemmung der intestinalen Motilität nach Ketamin‑/Midazolamnarkose. Ein Vergleich zur Narkose mit Enfluran und Fentanyl/Midazolam. Anaesthesist.

[65] Kissin I, Bright CA, Bradley EL (2000). The effect of Ketamine on opioid-induced acute tolerance: Can it explain reduction of opioid consumption with Ketamine-opioid analgesic combinations?. Anesth Analg.

[66] Nutt D, King LA, Saulsbury W, Blakemore C (2007). Development of a rational scale to assess the harm of drugs of potential misuse. Lancet.

[67] Ozkan A, Okur M, Kaya M, Kaya E, Kucuk A, Erbas M, Kutlucan L, Sahan L (2013). Sedoanalgesia in pediatric daily surgery. Int J Clin Exp Med.

[68] Wolff K, Winstock AR (2006). Ketamine: from medicine to misuse. CNS Drugs.

[1] Liu F, Paule MG, Ali S, Wang C (2011). Ketamine-induced neurotoxicity and changes in gene expression in the developing rat brain. Curr Neuropharmacol.

